# The global pattern of epiphytic liverwort disparity: insights from *Frullania*

**DOI:** 10.1186/s12862-024-02254-x

**Published:** 2024-05-14

**Authors:** Ying Yu, Mei-Ying Fan, Hong-Xia Zhou, Yue-Qin Song

**Affiliations:** College of Life and Environmental Sciences, Huangshan University, Huangshan, 245041 China

**Keywords:** Latitude disparity gradient, Morphospace, Ecological opportunity, Marchantiophyta, Paleo-environmental change, Efficient dispersal

## Abstract

The geographical and ecological patterns of morphological disparity are crucial to understand how species are assembled within communities in the context of the evolutionary history, morphological evolution and ecological interactions. However, with limited exceptions, rather few studies have been conducted on the global pattern of disparity, particularly in early land plants. Here we explored the spatial accumulation of disparity in a morphologically variable and species rich liverwort genus *Frullania* in order to test the hypothesis of latitude disparity gradient. We compiled a morphological data set consisting of eight continuous traits for 244 currently accepted species, and scored the species distribution into 19 floristic regions worldwide. By reconstructing the morphospace of all defined regions and comparisons, we identified a general Gondwana-Laurasia pattern of disparity in *Frullania*. This likely results from an increase of ecological opportunities and / or relaxed constraints towards low latitudes. The lowest disparity occurred in arid tropical regions, largely due to a high extinction rate as a consequence of paleoaridification. There was weak correlation between species diversity and disparity at different spatial scales. Furthermore, long-distance dispersal may have partially shaped the present-day distribution of *Frullania* disparity, given its frequency and the great contribution of widely distributed species to local morphospace. This study not only highlighted the crucial roles of paleoenvironmental changes, ecological opportunities, and efficient dispersal on the global pattern of plant disparity, but also implied its dependence on the ecological and physiological function of traits.

**Supplementary Information:**

The online version contains supplementary material available at 10.1186/s12862-024-02254-x.

## Introduction

The geographical and ecological patterns of morphological disparity are crucial to understand how species are assembled within communities in the context of evolutionary history, morphological evolution and ecological interactions, and to provide useful guidance for conservation purposes [1–4]. However, most geographical studies so far have biased towards the spatial patterns of species and ecological diversity [5–10] rather than towards disparity, i.e. the measure of morphological variation among species and higher taxa [11]. Thus, with a few exceptions for plants [12–17], we still do not have a good understanding of how morphological disparity accumulates along general environmental and latitudinal gradients, and what explains these patterns, particularly in land plants [2].

The latitudinal diversity gradient, i.e. increasing species diversity towards the tropics (LDG), is one of the most well-studied and recognized patterns in geography and ecology [5, 6, 8]. This pattern has been widely explored in land plant diversity [10, 18–21], and numerous hypotheses have been proposed to explain it, such as the dynamics of water-energy, habitat heterogeneity, and paleoenvironmental changes [22–25]. However, the generality of this geographical pattern in land plant disparity remains controversial [12, 14, 15]. Chartier et al. [12] has identified a latitudinal gradient in flower disparity, reflecting the release of biotic constraints and increase in plant-pollinator interactions towards low latitudes. However, this pattern was not supported in pollen disparity, largely due to phylogenetic conservation and/or adaptive evolution [15, 26]. Such inconsistency is in line with the expectations from recent studies [16, 17, 27–29], which have concluded that the recovered spatial trends of phenotypic disparity were not only affected by the complex of selective abiotic constraints, biotic interactions, and niche partitioning, but also depend on the ecological or physiological function of the traits studied. Currently, there is still insufficient evidence to draw conclusions, since such test was restricted to a few plant lineages and reproductive traits, e.g., pollen and flower traits [12, 15]. Thus, our knowledge of the latitudinal pattern of morphological disparity and relevant processes in plants is still poor, particularly in early land plants [2].

In this study we tested the “latitudinal disparity gradient pattern” (LDGP) in liverworts (Marchantiophyta) based on the insights from an epiphytic genus *Frullania* Raddi (Frullaniaceae, Porellales). *Frullania*, with ca. 350 species and a mainly tropical/subtropical distribution, represents one of the most species-rich leafy genera in liverworts [30–32]. These species are well defined by being dull green to brownish black plants, incubous leaves, a usually helmet-like leaf lobule and *Frullania*-type branching [33]. Apart from these taxonomic traits, this genus exhibits a rich diversity in morphology, particularly those related to plant and leaf size, such as stem width, and lobe and lobule size [33, 34]. Recent phylogenetic-based studies have greatly improved our understanding of the evolutionary history, taxonomy, ecology and geography of this genus [20, 35–38]. These new insights include: (1) *Frullania* was estimated to have originated in the lower Cretaceous (123 Ma) [37, 38], and experienced a burst of diversification since the Mesozoic, coinciding with the rise of forests dominated by angiosperms [35]. (2) Rampant homoplasy and rapid diversification are partly responsible for the extensive conflicts between the morphological and molecular phylogenies [30]. (3) Efficient dispersal does not obscure the pattern of a latitudinal gradient of epiphytic liverwort diversity [20, 36, 39, 40], considered as a result of ecological opportunity and climate change [20, 35].

Based on this previous work, we developed some hypotheses concerning the spatial evolution of *Frullania* disparity. Firstly, the tropics (excluding arid areas because of ecological stress and massive extinction, [7]) are expected to exhibit higher disparity than temperate regions. This is because of an increase of ecological opportunities and/or relaxation of ecological constraints, as well as the greater complexity of biotic interactions towards low latitudes [27, 35, 41]. Secondly, at a global scale, the correlation between species diversity and morphological disparity may be weak, given the difference in the evolutionary processes of both aspects in this group [42, 43]. Thirdly, efficient dispersion, particularly long-distance dispersal (LDD), is expected to have shaped the spatial pattern of disparity; for example, novel innovations will rapidly colonize new habitats following efficient dispersal. However, such effects may vary between terrestrial areas and islands, considering geographical isolation and dispersal limitations [44–46].

To test these hypotheses, we compiled a morphological dataset comprising of 244 currently accepted species and eight continuous traits. Using this dataset, we reconstructed the multi-dimensional morphospace of 19 defined geographical regions worldwide. In addition, we performed correlation analyses and measured the proportion of regional morphospace occupied by endemics in order to assess the effect of species diversity and LDD on the global pattern of morphological disparity.

## Materials and Methods

### Sampling and morphological trait data

The sampling includes 244 species (here we treated varieties and subspecies as species, [32], https://www.tropicos.org/home), covering ca. 70% of species diversity in *Frullania* (Table S1). These species were assembled to represent not only the taxonomic diversity [30, 32, 38], but also possible morphological and geographical variation observed in this genus [34, 47].

As the sporophyte data are inaccessible for most of species in this genus, we selected eight functionally significant and continuous gametophytic characters (Table S1), namely stem size (trait 1), lobe length and width (traits 2 and 3), lobule length and width (traits 4 and 5), under-leaf length and width (traits 6 and 7), and the ratio of lobule to lobe size (trait 8). The selected traits are involved in some key functions, such as water uptake and storage, and photosynthesis, that are crucial for taxa to survive and colonize in epiphytic habitats [48]. The values of eight selected traits were collected from taxonomical literature by estimating the middle of the maximum and minimum values.

### Geographical data

The distribution range of each species was obtained from the database of Winter [47] and our own observations on herbarium specimens (Table S1). We scored species distribution using the biogeographical scheme defined by van der Wijk et al. [49] and Tan & Pócs [50], with a few modifications to accommodate the biographical patterns observed in *Frullania*. In this scheme, 19 floristic regions were recognized (Fig. S1): Europe (EUR), northern Africa (AF1), continental sub-Saharan Africa (AF2), Mascarene Islands (AF3), southern Africa (AF4), northern Asia (AS1), eastern Asia (AS2), southern Asia (AS3), south-western Asia (AS4), western Asia (AS5), North America (AM1), Central America (AM2), Caribbean islands (AM3), northern South America (AM4), Brazil (AM5), southern South America (AM6), Australia (AU1), New Zealand (AU2), and Oceania (OC). This scheme has been widely used in recent geographical studied to describe the distribution of bryophytes [51, 52].

### Morphological disparity

The morphological data were log-transformed and then applied into the principal component analysis (PCA). The first five axes from the PCA, accounting for 95.84% of the total variance (Table S2), were extracted, and used to reconstructed 5-Dimension morphospace for the 19 defined geographical regions. All analyses were performed in SPSS (IBM Corp. Released 2017. IBM SPSS Statistics for Windows, version 25.0. Armonk, NY: IBM Corp.).

Morphological disparity for taxa in different floristic regions was estimated using the sum of maximum distance (MD). This index was calculated as the maximum uncorrected distance between species pairs using each component in a 5-D morphospace [e.g., MD_PC1_ = Max (PC1_taxonA_-PC1_taxonB_)]. Because five components scale differently in the available morphospace with respect to the evolutionary history, development processes and attributes of ecological niche space they aim to represent, we measured the sum of MD for each defined region using all five PC scores, e.g., MDAS1 = ∑MDPCi (AS1).

### The effects of species diversity and LDD

To test the relationship between morphological disparity and species diversity at different spatial scales, we performed Spearman correlations, taking the results of our pre-analyses into account. In these pre-analyses, both biodiversity aspects (species richness and morphological disparity) were logarithmically correlated. In addition, to reduce the effect of LDD, we performed an additional correlation analysis using a pruned sampling comprising of only endemics (referring to species exclusively occurring in one defined region).

Recent studies of population genetic structure suggested that the distance for efficient dispersal of bryophytes ranged from 100 m to 1 km, beyond which an increasingly small proportion of spores traveled [46]. However, it is still unfeasible to measure the frequency of LDD for a given bryophyte species in practice, as the present-day wide distribution could result from repeated efficient short-distance dispersal or several LDD events. Here we assumed that LDD more likely occurred for intercontinental distributed species, while less likely for endemics. To test this, we measured the proportion of local morphospace occupied by endemics. This index is expected to reflect the relative contributions from endemics and widely distributed species (referring species distributed two or more defined regions) to the whole regional morphospace. The higher the proportion is, the more contribution endemics have made, and the less effect LDD has imposed on the spatial accumulation of disparity.

## Results

**Fig. 1:**
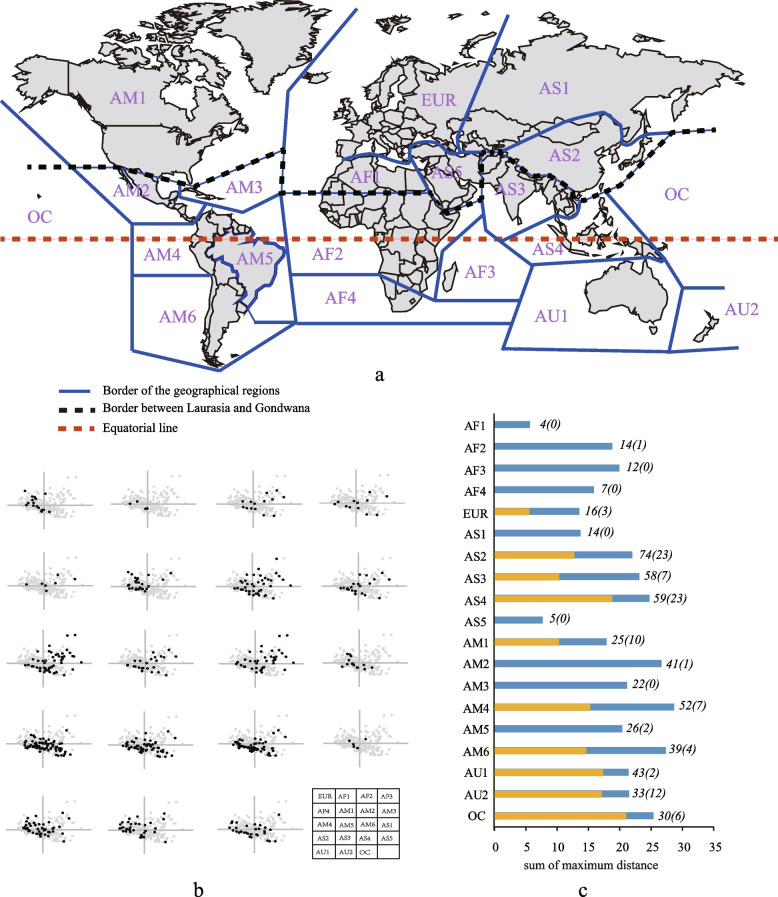
Pattern of *Frullania* disparity (the morphospace reconstructed by the first two PC components) and proportion of local morphospace occupied by endemic species (bars) in each of 19 world biogeographic regions defined by van der Wijk et al. [49] and Tan & Pócs [50]. a. The geographical scheme used in this study includes 19 regions: Europe (EUR), northern Africa (AF1), continental sub-Saharan Africa (AF2), Mascarene Islands (AF3), continental southern Africa (AF4), northern Asia (AS1), eastern Asia (AS2), southern Asia (AS3), southwestern Asia (AS4), western Asia (AS5), eastern North America (AM1), Central America (AM2), Caribbean islands (AM3), northern South America (AM4), Brazil (AM5), southern South America (AM6), Australia (AU1), New Zealand (AU2), and Oceania (OC). b. The regional morphospace reconstructed by the first two components PC1 and PC2. Both PC scores account for 81.97% of the total variance. Symbols represent 244 taxa in each graph. The dots in each figure were graphed using (*x*, *y*) with *x* corresponding to PC1 and *y* to PC2 scores. Black dots, species occurring in the highlighted geographical region; grey dots, all remaining sampled species. c. Morphological disparity and species diversity (endemic diversity in parentheses) in each 19 world geographical regions. Blue bars: the sum of the maximum distance using the first five PC scores and the original sampling. Yellow bars: the sum of the maximum distance using the first five PC scores and the pruned sampling with inclusion of exclusively endemics

By reconstructing 5-D morphospace for 19 geographical regions worldwide and comparing them, we identified a general Gondwana-Laurasia pattern of disparity distribution in *Frullania* (Fig. 1, Fig. S1, S2). In it, Gondwanan regions show relatively higher disparity than Laurasian regions, except for east Asia (AS2), which is part of Laurasia but exhibits a similar level of disparity to most of Gondwana’s (MD_AS2_ = 22.08, Fig. 1). The highest disparity occurs in Central and South America (AM2, AM4, and AM6, all with a MD > 25, Fig. 1, Fig. S1, S2), while a relatively lower disparity occurs in arid tropical areas, including west Asia (AS5) and North Africa (AF1) with a MD < 10 (Fig. 1, Fig. S1, S2).

There is a significant correlation between species diversity and disparity using the original sampling (Spearman correlation r = 0.751, *p* < 0.001), but no a correlation using the pruned sampling (including only endemics, r = -0.286, *p* = 0.456). A survey of geographical distributions discovered that almost 50% of the samples (125 of 244 species) were distributed across two or more geographical regions, and among them, ca. 40% (50 of 125 species) had inter-continental distributions (Table S1, Fig. S3). At the regional scale, more than 53% of the local diversity are widely distributed species. Among all defined regions, endemics account for 0% to 85% of the regional morphospace (Fig. 1). Notably, the proportion is much higher in islands (> 70%, e.g., AU1, AU2, OC and AS4, Fig. 1) than in the mainland regions (< 60%, Fig. 1).

## Discussion

The recovered geographical pattern of morphological disparity in the liverwort genus *Frullania* is partly consistent with the LDGP hypothesis, which predicts an increase of disparity towards low latitudes. Such pattern also highlights the variability of morphological disparity across tropical regions, that is, both the highest and lowest disparity occurring in tropics (Fig. 1). One possibility for this complex pattern is ecological opportunity or ecological constraints. In this case, the evolution of selected traits is expected to be influenced by some climatic factors that are related to water-availability [53, 54], concerning their ecological functions, e.g., uptake of water and mineral nutrition from the surrounding air [48, 55]. Therefore, the higher disparity of epiphytic liverworts at low latitudes may be related with an increase of ecological opportunities provided by tropic and subtropic forests, e.g., rich diversity of vascular plants as epiphytic substrates and extensive resources [41, 56], and / or the relaxed ecological constraints, e.g., high humidity [34, 53], and / or phenotypic innovation that facilitate ecospace expansion [13, 57, 58]. This assumption can also be used to explain the observations in some temperate regions, e.g., East Asia, which also exhibit high diversity and disparity (Fig. 1), due to availability of novel ecological opportunities in these regions that are provided by mountains and other complex topographic characteristics, e.g., deeply dissected plateaus [59–62]. In contrast, the lowest disparity in the arid tropical area may be largely due to the higher extinction rate, caused by the massive loss of the rainforest area as a consequence of sustained aridification during the Cenozoic [7, 63].

Our correlation analyses indicated that the effect of species diversity on the spatial pattern of disparity may be limited, for two reasons. Firstly, a statistically significant correlation between diversity and disparity using the original sampling was probably biased by LDD. This is because some widely distributed species are positioned at the periphery of 5-D morphospace, leading to the regions spanned by these species tending to have a similar level of disparity in spite of the difference in species diversity, e.g., AM3-5 (Fig. 1). This view was supported by disparity estimation and additional correlation analyses, in which the local morphospace of all defined regions decreased by about 20–100%, and there is no longer a correlation between diversity and disparity using the pruned sampling.

Furthermore, rapid speciation is not always accompanied by morphological differentiation. Taking East Asia (AS3) as an example, this region harbors a great diversity of anciently and recently originated bryophytes and vascular plants, largely due to recent rapid diversification caused by historical orogenesis and the consequent environmental changes [64, 65]. This scenario was also found in *Frullania*, in which species tend to be close to the centre of the present-day morphospace (Fig. 1), implying recent rapid speciation but slow phenotypic differentiation. This pattern has been repeatedly recovered in the phylogenetic trees of land plants [66] and animals [67], and is usually considered as a result of evolutionary rate heterogeneity [68, 69]. However, we cannot rule out the possibility that both diversity and disparity have been shaped by the same factors, such as ecological opportunity, which in empty form (newly formed and / or opened ecospace due to evolutionary innovations) is expected to promote both diversification and differentiation [70].

LDD has long been considered as a key factor shaping the geographical assembly of bryophyte diversity [29, 44–46, 71, 72]. In the case of *Frullania*, this view is supported on the basis of species richness and disparity. Although the spatial pattern of species richness was not explored in detail in this study, a survey of species distribution indicated that nearly half of *Frullania* species, and more than 53% of local diversity are widely distributed (Fig. 1), suggesting an important role of LDD. Nevertheless, our understanding of how LDD affects the geographical assembly of species diversity at the global scale remains poor, because of a lack of research on the history and frequency of these LDD events. In parallel, widely distributed species accounted for 15–100% of local morphospace across 19 defined regions, implying that LDD has partly shaping the present-day global pattern of disparity. Furthermore, this proportion is much higher in continental areas than in islands (Fig. 1), suggesting geographical barriers [73, 74], dispersion limits [45, 46], and/or the reduced influence of this factor on the saturation of local morphospace, which tended to happen very early in the evolution of clades [75, 76].

## Conclusion

This study not only recovered a complex distribution pattern of liverwort disparity at the global scale, i.e. it varies between Gondwana regions (mostly tropical and subtropical) and Laurasia’s (mostly temperate), combined with “tropical morphological diversity disparity”, but also highlighted the crucial roles of paleoclimate changes, ecological opportunity, and LDD. The former two factors may have been involved in defining available morphospace [61, 75, 77, 78], and LDD may accelerate morphospace occupation early in the evolution of clades. Although this work fills a gap in our understanding of the spatial evolution of morphological disparity in early land plants, there are still many questions that are needed to be addressed in future studies using a large sampling and comprehensive phylogenetic-based framework, such as: how does morphological disparity respond to paleo- or present climate and geographical changes? Is the disparity correlated with the duration of lineages or geographical regions? In addition, this work reignites people’s concern about the destruction of forest ecology caused by climate change and human activities, which leads to the reduction of ecospace of these small and vulnerable liverworts, and subsequently biodiversity loss. This ongoing reality urges researchers to accelerate the study of the geographical patterns of bryophyte biodiversity, which could provide scientific biases for the development of practical conservation strategies, with particular focus on the forest in East Asia and islands that embrace the greatest biodiversity and endemics.

### Supplementary Information

Supplementary Material 1.

Supplementary Material 2.

## Data Availability

All data generated during this study are included in this published article and its supplementary information files.
