# Supplementary material for: The global pattern of epiphytic liverwort disparity: insights from Frullania
Source: BMC Ecol Evol. 2024 May 14;24:63. doi: 10.1186/s12862-024-02254-x (PMC11092184; doi:10.1186/s12862-024-02254-x)
Supplement: Supplementary file 2 — Supplementary Material 2. [file 12862_2024_2254_MOESM2_ESM.pdf]

Supplementary (Fig. S1-S3)

Article Title：The global pattern of epiphytic liverwort disparity: insights from *Frullania*

Journal: BMC Ecology and Evolution

Authors: Ying Yu*, Mei-Ying Fan, Hong-Xia Zhou, Yue-Qin Song

Affiliation: College of Life and Environmental Sciences, Huangshan University, Huangshan 245041, China

***Author for correspondence: yuying@hsu.edu.cn**

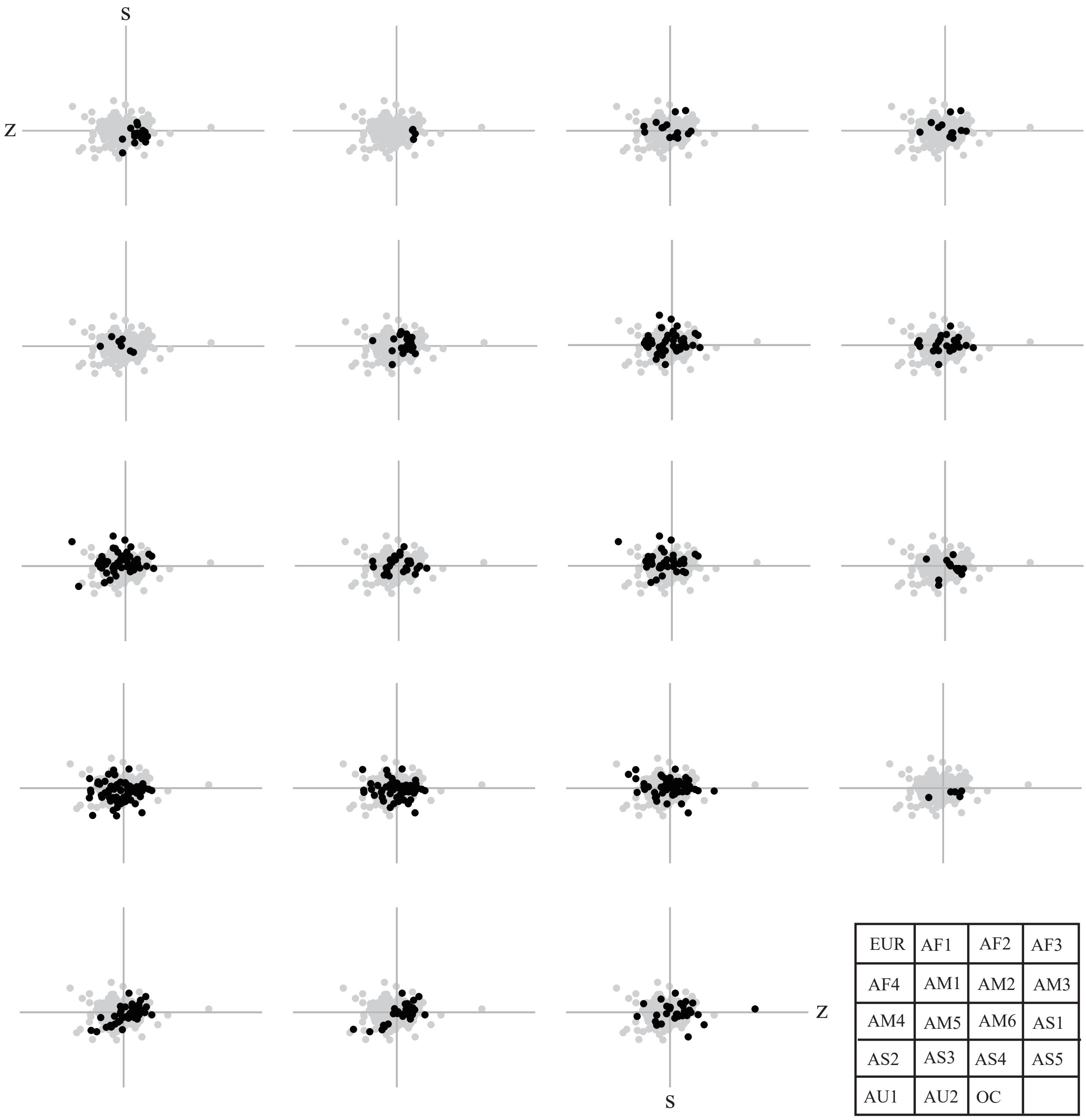

Figure S1 The regional morphospace of *Frullania* reconstructed by the third and fourth components PC3 and PC4. PC3 and PC4 account for 5.60% and 4.50% of the total variance respectively. Symbols represent 245 taxa in each graph. The dots in each figure were graphed using (z, s) with z corresponding to PC3 and s to PC4 scores. Black dots, species occurring in the highlighted geographical region; grey dots, all remaining sampled species.

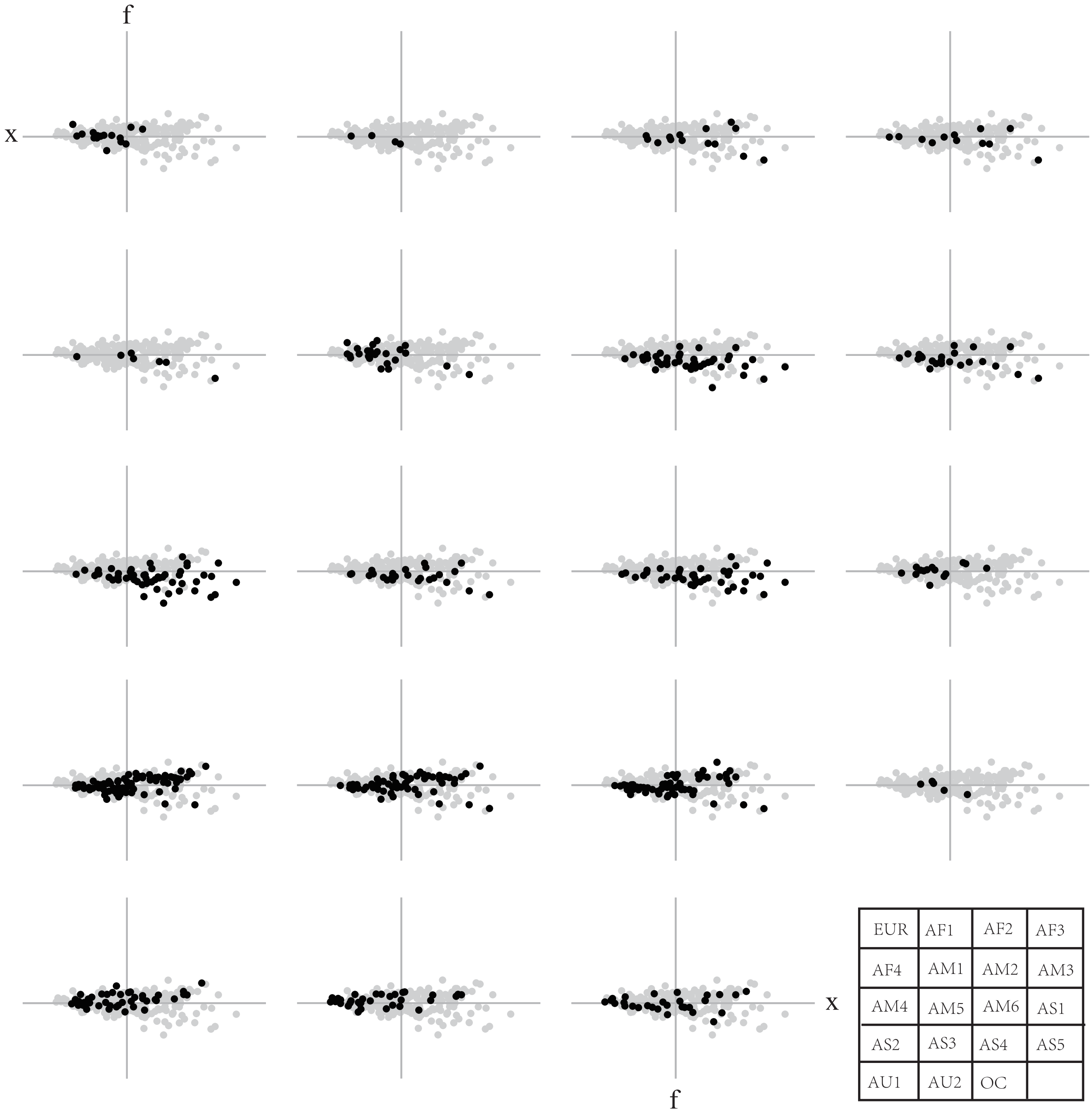

Figure S2 The regional morphospace of Frullania reconstructed by the first and fifth components PC1 and PC5. PC1 and PC5 account for 59.81% and 3.78% of the total variance respectively. Symbols represent 245 taxa in each graph. The dots in each figure were graphed using (x, f) with x corresponding to PC1 and f to PC5 scores. Black dots, species occurring in the highlighted geographical region; grey dots, all remaining sampled species.

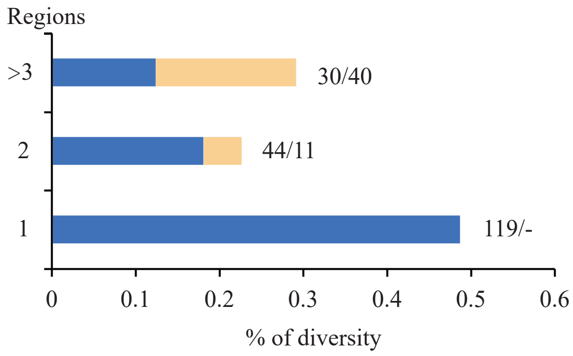

Figure S3 Summary of geographical distributions of 244 species sampled. The colored bars represent the ratio of diversity. Blue, species occurring within one continent; yellow, species occurring in two or more continents. The corresponding numbers of species are listed beside the bars.
